# Modulators of Oxidative Stress: Chemical and Pharmacological Aspects

**DOI:** 10.3390/antiox9080657

**Published:** 2020-07-24

**Authors:** Luciano Saso, Hande Gürer-Orhan, Višnja Stepanić

**Affiliations:** 1 Department of Physiology and Pharmacology “Vittorio Erspamer”, Sapienza University, 00185 Rome, Italy; luciano.saso@uniroma1.it; 2 Toxicology Department, Faculty of Pharmacy, Ege University, Izmir 35040, Turkey; hande.gurer.orhan@ege.edu.tr; 3 Ruđer Bošković Institute, Bijenička cesta 54, 10000 Zagreb, Croatia

Oxidative stress is represented as an imbalance between reactive oxygen species (ROS) production and the response of antioxidant proteins. Oxidative stress is implicated in the pathogenesis of a variety of conditions, including cardiovascular diseases, neurodegenerative diseases, and cancer. Unfortunately, antioxidant therapies have not been proven to be effective in most clinical studies [1]. One of the reasons for this disappointing failure was testing antioxidant potential of compounds only through their capacity to reduce the number of free radicals directly by their free radical scavenging and metal chelating activities. It took some time to realize that some small molecules can achieve a more significant antioxidant effect by targeted modulation of activities of proteins included in the antioxidant system [2]. Relatively recently modulation of oxidative stress through regulation of gene transcription has been discovered to be a promising strategy for the development of new drugs for cardiovascular diseases and cancer types that are resistant to other treatments [3]. This Special Issue of the “Antioxidants” collects seven original research manuscripts and six reviews addressing different pharmacological aspects of the modulation of oxidative stress by natural and synthetic small molecular weight compounds in regard to various therapeutic approaches in cardiovascular and neurodegenerative diseases, cancer and diabetes. Understanding the relationship between oxidative stress and underlying anti- and pro-oxidant mechanisms is crucial to understand mechanisms of diseases and also for the discovery and development of innovative, targeted therapeutic strategies.

The two research articles present the results of cellular studies on contribution of antioxidants in alleviating ischemia–reperfusion (IR) injuries in myocardial cells and cardiac fibroblasts (CF). IR injury is a common clinical problem that occurs in the heart (in myocardial infarction) as well as other organ systems (e.g., in the brain in stroke and in limb ischemia) and may also be a result of reperfusion strategy or occur during surgeries [4]. IR occurs when blood supply to an organ is diminished and then re-established. Although blood flow is necessary for survival, reperfusion induces necrotic and apoptotic cell death. One of the key mediators of reperfusion injury is an oxidative stress. A huge generation of ROS at the beginning of reperfusion initiates lipid peroxidation and protein/nucleic acid oxidation, consequently activating pro-apoptotic pathways associated with p38-MAPK and JNK proteins [5]. In their manuscript, Parra-Flores et al. [6] showed for the first time synergistic cytoprotective effect of ascorbic acid, deferoxamine, and *N*-acetylcysteine in simulated IR in CF. When added at low concentrations (10 μM) each at the onset of simulated reperfusion, the three antioxidants synergistically reduced intracellular ROS production and increased cell viability, induced phosphorylation of pro-survival kinases ERK1/2 (extracellular-signal-regulated kinases 1/2) and PKB (protein kinase B)/Akt, and decreased phosphorylation of the pro-apoptotic kinases p38-MAPK (proteins p38-mitogen-activated protein kinase) and JNK (c-Jun-N-terminal kinase). The mixture also recovered the CF function associated with wound repair by restoring serum-induced migration, TGF-1-mediated differentiation of CF into CMF and angiotensin II-induced pro-collagen I synthesis. The three compounds have different mechanism of antioxidant activity and act at concentrations higher than 10 μM. However, in the mix, the oxidized form of ascorbate—dehydroascorbate, can be transformed back in the reduced form by intracellular glutathione (GSH) [7] and the decreased intracellular concentration of GSH may be replenished by *N*-acetylcysteine which is a GSH precursor. Deferoxamine can chelate an excess of catalytic free iron which increases during ischemia and cardiac reperfusion from cell lysis, and in this way the production of OH radicals through the Fenton reaction is lowered, thus preventing the pro-oxidant interaction of iron with ascorbate [8]. In another study, Peserico et al. [9] pointed to the repurposing of ezetimibe from a lipid-lowering compound to the antioxidant with diminishing effects of the oxidative stress induced by the simulated IR in the human monocyte-like cells THP-1 and cardiomyocytes [10]. Overnight pre-incubation of the cells with ezetimibe (50 μM) reduced ROS formation, NF-kB activation and apoptosis, induced by simulated IR. Ezetimibe induced up-regulation of NRF2 (Nuclear factor erythroid 2-related factor 2)/ARE (antioxidant response element) signalling and the pro-survival UPR (unfolded protein response) pathway by up-regulation of the pro-survival activating transcription factor 6 (ATF6) and down-regulation of the pro-apoptotic CCAAT-enhancer-binding protein homologous protein (CHOP). In such a way, misfolded protein mass may be reduced and cells convert to a pro-survival state. The NRF2 activation was found to be dependent on AMPK phosphorylation.

Oxidative stress and depletion of intracellular antioxidants, together with a systemic inflammatory response and ultimately organ failure are characteristics of the sepsis [11]. Since organ dysfunction associated with sepsis is due, at least in part, to mitochondrial dysfunction and mitochondrial dysfunction is considered as main cause of oxidative stress in sepsis, antioxidants targeted to mitochondria have been considered as an efficient antioxidant protection in sepsis [12]. Minter et al. [13] compared the positively charged antioxidant MitoVitE, which accumulates within mitochondria, with the membrane-bound α-tocopherol and water-soluble cytosolic Trolox with regard to their effects on oxidative stress, mitochondrial function and expression of key genes and proteins involved in the toll-like receptor (TLR)-2 and -4 inflammatory signalling pathways in the umbilical vein endothelial cell line HUVEC-C under conditions mimicking acute bacterial sepsis. Each of these three forms of vitamin E (5 μM each) was found to have similar reducing effects against induced oxidative stress and NFκB activation caused by exposure to lipopolysaccharide/peptidoglycan G. However, in difference to α-tocopherol and Trolox, MitoVitE had also widespread downregulatory effects on gene expression involved in TLR pathways, resulting in anti-inflammatory profile. Many of these downregulated genes have been shown to be upregulated in mononuclear cells from patients with sepsis.

Mitochondrial dysfunction and consequential oxidative stress are also connected with organ injuries caused by exposure to ionizing radiation in cancer therapy [14]. A serious limitation to radiation therapy for some cancers is the possibility of irradiating the liver and induced liver injury. When cells are exposed to radiation, the redox system begins producing free radicals a few hours after exposure, but with the potential to last for years and cause pathological long-term persistent liver responses to ionizing radiation, such as radiation-induced liver disease (RILD) [15]. It has been known that major mitochondrial deacetylase sirtuin 3 (SIRT3) contributes to the development of ionizing radiation-induced acute liver injury [16]. The results of the in vivo study conducted by LoBianco et al. [17] show that loss of or decrease in SIRT3 levels could be an important factor contributing to a damage-permissive phenotype in murine liver long after exposure to ionising radiation. Their study on the two groups of Sirt3−/− and Sirt3+/+ male mice six months after their liver was exposed to 24 Gy radiation additionally suggested that superoxide radical anion-driven acute liver injury following radiation exposure appears to shift towards a peroxide-mediated long-term injury and is no longer limited to the mitochondria. On the other hand, there is a possibility to use radiation to stimulate cellular mechanisms and facilitate the removal of oxidative stress through the modulation of various signalling pathways in the presence of nonionizing light at a specific wavelength. In their review article, Rajendran et al. [18] described and discussed the effects of photobiomodulation on the progress of cancer, diabetes and wound healing through the crosstalk between ROS and the first discovered redox-regulated transcription factor, NF-кB (nuclear factor kappa-light-chain-enhancer of activated B cells).

A master regulator of cellular redox homeostasis is the transcription factor NRF2 [1,3]. NRF2 is a regulator of the transactivation of over 500 cytoprotective genes, many of which are included in the antioxidant defence against intracellular ROS increase. The major signalling pathway that regulates oxidative stress is NRF2–KEAP1 (Kelch-like ECH-associated protein 1). Panieri et al. [19] reviewed the activation mechanisms and the therapeutic modulation of NRF2 within the specific biological context. In the early stages of carcinogenesis, pharmacological induction of the NRF2 pathway might be chemopreventive. The NRF2 inducers protect normal cells from carcinogen effects by reducing the risk of cancer initiation and development in normal cells by ROS scavenging, decreasing DNA damage and preventing genomic instability. However, in advanced stages of cancer, the adverse effects of the NRF2 induction might occur due to the development of therapy resistance [20]. Over-activation of the NRF2–KEAP1 pathway in many tumours has been found to promote cancer growth and survival, metastasis formation and therapy resistance. Sustained NRF2 activation in cancer cells lead to metabolic changes that frequently develop “NRF2 addiction” [21]. Panieri et al. pointed to disruption of NRF2 signalling in cancer cells by using NRF2 inhibitors as a promising therapeutic approach against NRF2-addicted cancers. 

NRF2 activators have also been proposed as antioxidants in neurodegenerative and neuropsychiatric disorders (Alzheimer’s disease, Parkinson’s disease, Huntington’s disease, autism, schizophrenia, and depression) to counteract the increase in oxidative stress and inflammation. Alvarez-Arellano et al. [22] made an overview of the role of inflammation and oxidative stress in attention-deficit/hyperactivity disorder (ADHD). The amelioration of inflammation and oxidative stress by natural antioxidants sulforaphane, *N*-acetylcysteine and omega-3 fatty acids (docosahexaenoic acid (DHA) and eicosapentaenoic acid (EPA)) in potential adjuvant therapy of ADHD is described, and the observed effects of these antioxidants on the activation of the NRF2 pathway in neurodegenerative and neuropsychiatric disorders are summarized.

García-Guede et al. [23] reviewed the effects of oxidative stress on the epigenetic machinery of DNA methylation, histone modifications and non-coding RNA, particularly microRNAs (miRNAs) and long non-coding RNAs (lncRNAs). The influence of these epigenetic modifications is extensively described in the case of the specific mechanisms for developing cisplatin resistance.

In the four studies reported in this Special Issue, the effects of food components on human health through modulation of oxidative status of the body cells are investigated. One of the irreplaceable food component necessary for maintaining health and preventing diseases for humans is ascorbic acid/vitamin C [24]. By employing untargeted mass spectrometry and metabolomics analysis of the C2C12 myoblast cells pre-treated with ascorbic acid (100 µM), Magaña et al. [25] suggested that co-supplementation of ascorbate with folic acid may activate the folate-mediated one-carbon cycle, thereby promoting the conversion of homocysteine into methionine while reducing cellular oxidative stress. The pre-treatment of the C2C12 myoblast causes a change in the concentration of tetrahydrofolate (THF) metabolites. Ascorbate appears to spare NAD(P)H and thus increases availability of reducing equivalents for the reductive steps from 10-formyl-THF to 5-methyl-THF via 5,10-methylene-THF. In agreement, an increase in the levels of methionine, whose formation from homocysteine is 5-methyl-THF dependent, and an increase in thymidine, whose formation from deoxyuridine monophosphate (dUMP) is dependent on 5,10-methylene-THF, were observed. 

Castejón et al. [26] made a comprehensive review of secoiridoid compounds of the olive tree (*Olea europaea* L. (Oleaceae)) in regard to the chemical, biosynthetic and pharmacological (including pharmacokinetic) aspects. They have tabulated and discussed the effects of the main olive phenolics, namely, oleuropein and its aglycon, oleocanthal and oleacein, which were observed recently in in vitro and in vivo studies in relation to the control and progression of different types of cancer, cardiovascular diseases and neurodegeneration processes in autoimmune diseases, as well as their potential roles in anti-aging.

Overconsumption of some type of food has been associated with the induction of oxidative stress and consequentially the development of pathological transformations. In the review conducted by Cherkas et al. [27], current epidemiological evidence and redox biology advancements are discussed with the purpose of defining the linkage between the observed disruptive metabolic effects, detrimental health consequences of chronic nutritional carbohydrate overload and the metabolic role of glucose in maintaining redox homeostasis. Glucose is the most important energy source and glycolysis is a main pathway for obtaining energy when glucose is abundant and redox conditions are favourable. The authors hypothesized that under extreme oxidative and toxic challenges, glucose provides stress resistance by the reduction of nicotinamide adenine dinucleotide phosphate NADP^+^ to NADPH through its metabolism in the pentose phosphate pathway (PPP). The reduced form NADPH is needed for the reduction of oxidized glutathione and protein thiols, the synthesis of lipids and DNA as well as for xenobiotic detoxification, regulatory redox signalling and counteracting infections. In regard to this aspect, they discussed the role of glycogen stores in resistance to oxidative challenges. 

The important approach to study ageing and age-linked pathologies is to investigate sex dimorphism in the defence against metabolic stressors. In most mammals, females show lower incidence of some age-related pathologies linked with oxidative stress conditions, and this sex difference disappears after menopause, which led to the conclusion that this protection is attributed to the effect of sex hormones [28]. By conducting in vivo experiments on Sirt3 wild-type (WT) and knockout (KO) mice of both sexes fed with a standard or high-fat diet (HFD) for ten weeks, Pinterić et al. [29] found significant sex differences in male and female mice responses at the metabolic level, oxidant/antioxidant status, and liver mitochondrial parameters. Their study points towards a different role of SIRT3 in males and females under the conditions of nutritive stress. For example, the male-specific effects of an HFD include reduced SIRT3 expression in WT and alleviated lipid accumulation and reduced glucose uptake in KO mice.

The research articles and reviews collected in this Special Issue clearly contribute to further appreciation and enlightening of the underlying complexity of the investigation of chemical and pharmacological aspects of modulators of oxidative stress and the importance of studying the modulation of NRF2–KEAP1 and other relevant signalling pathways in order to develop effective drugs for diseases in which oxidative stress plays a significant etiopathogenetic role.
